# The influence of perceived causation on judgments of time: an integrative review and implications for decision-making

**DOI:** 10.3389/fpsyg.2013.00217

**Published:** 2013-05-14

**Authors:** David Faro, Ann L. McGill, Reid Hastie

**Affiliations:** ^1^London Business SchoolLondon, UK; ^2^University of Chicago Booth School of BusinessChicago, IL, USA

**Keywords:** time perception, causality, intentional binding, placebos, intertemporal choice, planning fallacy, judgment and decision making, agency

## Abstract

Recent research has shown that the perception of causality affects the judgment of elapsed time: an interval between an action and a subsequent event seems to be shorter when people believe that action has caused the event. This article reviews past work on the phenomenon and integrates the findings from the different settings in which it has been observed. The effect is found for actions people have personally taken, as well as for those they have simply read or heard about. It occurs for very short intervals (e.g., milliseconds) as well as longer periods (e.g., months or years). Beliefs and expectations about different types of causal forces and their trajectories over time can affect the degree of time compression in some settings. But the tendency toward compression of time is the default and dominant response: It persists when people think of generic causal relations and is enhanced when people opt for the quickest interpretation of causal relations. This robust influence of causality on time judgment appears to be linked to the basic tendency to rely on temporal proximity in processing causal relations and to people's early experience with the physical-mechanical world. Past work has focused primarily on the implications of time compression for the sense of agency, but this phenomenon has implications also for decisions that depend on time judgment. The compression of subjective time elapsed between actions and outcomes makes people more optimistically plan the timing of a focal action in the future, experience its effect earlier in the future, and be less likely to switch to an alternative course of action. The tendency toward compression can thus endow an action with a sort of privileged status or advantage.

Time plays an important role in causal inference. People generally expect a short time to have elapsed between causes and their effects and so rely on time as a cue to causation, judging an event that occurred closer in time to an effect as a more likely cause than one that occurred at a greater temporal distance (Hume, 1938; Michotte, 1963; Einhorn and Hogarth, 1986; Shanks et al., 1989; Lagnado and Sloman, 2006). Recent work has demonstrated, however, that this relationship between time and causality is bidirectional—the perception of causality can affect people's judgments of time in such a way that they perceive events that they know to be causally related to have occurred closer in time to each other.

For example, one set of studies focused on intentionality and showed that perceived time between a voluntary movement (e.g., pressing a key) and its effect (e.g., an auditory tone) was shorter compared to a baseline condition in which the action and its effect occurred within the same amount of time but without any causal link (the *intentional binding* effect; Haggard et al., 2002; see also Eagleman and Holcombe, 2002; Wohlschlager et al., 2003; Engbert and Wohlschläger, 2007; Moore et al., 2009; Ebert and Wegner, 2010). Other researchers focused on causality more generally and contrasted, for instance, estimates of time elapsed between pairs of historical events that were causally related (e.g., the launch of Sputnik by the USSR and the landing of man on the moon with Apollo 11) with estimates of time between historical events that were not causally related (e.g., the launch of Sputnik and the Woodstock music festival; Faro et al., 2005; see also Buehner and Humphreys, 2009; Faro, 2010; Faro et al., 2010; Buehner, 2012). Here, too, perceived causality shortened estimates of elapsed time.

In this article we review some of the findings from the different settings in which an influence of causality on time judgments has been observed.^1^ We have two primary goals. The various lines of work examining this phenomenon have proceeded mostly independently, but there are links among the findings that may shed light on the general tendency and why it may occur. We draw attention to these. Our second goal is to divert some of the focus from agency to time perception. Most of the work in this area has focused on the implications of the effect for the sense of agency. In particular, because compression of time was not observed for involuntary movements, researchers suggested the effect may be an implicit marker of agency (Haggard et al., 2002; see Moore and Obhi, 2012, for a recent review on the link between this phenomenon and agency). In this article we focus primarily on the implications that the effect has for decisions that depend on the judgment of time. The compression of subjective time elapsed between actions and outcomes makes people more optimistically plan the timing of a focal action in the future, experience its effect earlier in the future, and be less likely to switch to an alternative course of action. The tendency toward time compression can thus endow an action with a sort of privileged status or advantage (see also Engbert and Wohlschläger, 2007).

Our review proceeds as follows. First, we examine the robustness of the effect in different settings. We then review some process evidence, focusing particularly on several points of linkage between those findings that may help explain why the effect occurs and why it seems to be so general. Finally, we review the implications of the phenomenon.

## The generality of the effect

Early studies have shown an effect of perceived causation on time judgment by varying the intentionality of motor movements (Haggard et al., 2002; see also Wohlschlager et al., 2003; Engbert and Wohlschläger, 2007; Moore et al., 2009; Ebert and Wegner, 2010). Most of these studies used the Libet clock method, in which participants watch a rotating clock and report the position of the clock hand to indicate the onset of certain events they experience (Libet et al., 1983). In the baseline conditions of a study by Haggard et al. (2002), some participants voluntarily pressed a key while others heard an auditory tone. They indicated the timing of these events on the rotating clock. In the experimental conditions, participants indicated the timing of pressing the key or hearing the tone, but here the tone followed their action. The authors found that in the experimental conditions the perceived time of action was later than the baseline condition and the perceived time of the outcome was earlier than in the baseline condition. Thus, intentionality compressed the time interval between actions and their outcomes. In another set of conditions that tested whether intentionality indeed drives the compression of time, involuntary movements were induced in participants via transcranial magnetic stimulation. Haggard et al. found that in these conditions the compression effect was reversed such that the interval between involuntary action and effect was longer relative to the baseline.

While this initial set of studies focused on intentionality of action, later studies manipulated causality per se. For instance, some studies manipulated perceptions of causation apparent in historical events participants read about (Faro et al., 2005). Other studies have shown the effect of causation on time judgment through perceptual methods using Michotte's launching paradigm (Cravo et al., 2009), by manipulating the experienced covariation or probability of yielding the outcome (Engbert and Wohlschläger, 2007; Moore et al., 2009), and by providing study participants an alternative cause that discounts the role of a focal cause post experience (Faro, 2010). These different demonstrations have shown that the phenomenon is robust to different conceptualizations of causality. They also illustrate that the effect can occur irrespective of when causality is manipulated. Time between actions and outcomes seems shorter when causality is “sensed” before the time interval and the outcome, when it is learned during the repeated experience of the action-outcome sequences, and when casual beliefs are acquired after the experience of the events and the interval.

Various operationalizations of the dependent measure (elapsed time) have been employed, including a derived interval from perceived times of occurrence using the Libet clock method (Haggard et al., 2002; Wohlschlager et al., 2003; Engbert and Wohlschläger, 2007; Moore et al., 2009), a direct estimate of elapsed time on a unit scale (Faro et al., 2005; Engbert et al., 2008; Humphreys and Buehner, 2009; Moore et al., 2009), and reproduction of the experienced interval (Faro, 2010; Humphreys and Buehner, 2010). Finally, the effect has been demonstrated using a variety of timeframes and action-outcome sequences including motor movements and outcomes separated by milliseconds (e.g., Haggard et al., 2002), actions and outcomes separated by longer intervals of up to 4 s (e.g., Humphreys and Buehner, 2009), consumption of a product and the experience of its effect minutes later (Faro, 2010), and pairs of historical events that are years apart (e.g., Faro et al., 2005). Table 1 provides information about different manifestations of this effect.

**Table 1:** **Summary of some studies showing an effect of causality on time judgments**.

**References**	**Experimental task (operationalization of cause and effect)**	**Method of interval assessment**	**Range of studied time intervals**	**Proposed process/explanation**	**Proposed consequences/implications**
Buehner and Humphreys, 2009; Humphreys and Buehner, 2009	Press key—hear auditory tone	Numeric estimates, event synchronization	150 ms–4 s	Priming of general causality-time relationship	Anticipated action timing
Ebert and Wegner, 2010	Pull/push joystick—see object move on	Numeric estimates	100–700 ms	Retrospective inference	Binding associated with explicit sense of authorship
Engbert and Wohlschläger, 2007	Press key—hear auditory tone	Libet clock method	250 ms	Predictive motor process based on expectations and perceptual associative process	Priviledged representation of intentional actions
Faro, 2010	Take energy product—feel enhanced alertness	Numeric estimates, reproduction	38 s–6.5 min	Retrospective inference based on general causality-time relationship	Delayed consumption, early experience of effect, reluctance to switch to alternative actions
Faro et al., 2005, 2010	Sputnik launch— Apollo 11 landing (historical events)	Numeric estimates	3–184 years	Retrospective inference based on physical-mechanical causality	Evaluation of actions undertaken by others
Haggard et al., 2002	Press key—hear auditory tone	Libet clock method	250–600 ms	Predictive motor control process linking intentional actions and their outcomes	Coherent experience of agency, early experience of effect
Moore and Haggard, 2008; Moore et al., 2009	Press key—hear auditory tone	Libet clock method, numeric estimates	100–700 ms	Predictive motor control process and retrospective inference	Coherent experience of agency, early experience of effect

The generality and robustness of the effect of causality on time judgments is noteworthy because time perception phenomena are known to be context dependent; effects that were found in one setting were often not observed in others (Block and Zakay, 1997; Tourangeau et al., 2000). Further, the effect occurs both in settings in which people are likely unaware of any notions of causality per se, as well as in settings in which causality and judgments of time are more explicit in the experimental setting. Irrespective of the setting, the tendency for compression is the common default, and it is the standard finding in studies examining causality and time. These consistent findings suggest that the effect is strong, robust, and may reflect a basic tendency in the way people treat causality and time. In the following sections we review some research that may suggest why this may be the case.

## Explanations and interpretations

The initial findings of Haggard et al. (2002) on intentional binding were seen as evidence for a predictive motor-control process in the brain that adjusts the perceived timing of voluntary actions and their effects and provides a coherent experience of agency. The findings were also interpreted from a Bayesian perspective as the inverse of the Humean notion of temporal proximity's being a cue to infer causality. In particular, if people tend to attribute causal relations to events that are close to each other in time, then, under uncertainty about time, people may shift their estimates of time for causes and effects toward each other (Eagleman and Holcombe, 2002; see also Buehner and Humphreys, 2009). This argument is related to the notion of attribute substitution, whereby a variable that is hard to judge (e.g., time) may be replaced by a correlated variable (e.g., causality) to which a person may have easier access (Kahneman and Frederick, 2002). This Bayesian interpretation of the phenomenon as an automatic response that relies on a relationship between two correlated variables suggests that factors that promote reliance on correlated cues and shortcuts may moderate the effect. In line with this, cognitive load, for instance, resulted in greater compression of subjective time between causally related historical events (Faro, 2010; Faro et al., 2010). Thus, with motor movements as well as with more-conceptual action-outcome sequences, the compression effect was seen as an automatic brain response or judgment that relies on the general relationship between time and causality (Table 1).

Later work on this phenomenon has suggested that it can also be driven by inferential processes. In one study, Moore and Haggard (2008) manipulated the probability (50% vs. 75%) that the action (press of a key) resulted in the outcome (the tone). The authors found that when the action was unreliable in causing the outcome (in the 50% condition), there was time compression only in the trials in which the outcome occurred. In contrast, the occurrence of the outcome did not have a significant effect on time compression when the action was a more reliable predictor of the outcome (in the 75% condition). This was interpreted as evidence that time compression can occur through a retrospective inference process, because whether the outcome occurred was known only after the fact (after the action and the time interval). In a parallel finding, though in a very different setting, retrospective expert information that two historical events were causally related compressed time estimates, but only when the causal relationship between the two events was ambiguous (Faro et al., 2005). This suggests that predictive (pre-experience) as well as retrospective cues to causality can result in time compression and that they can substitute for each other (Moore et al., 2009)

The findings that retrospective cues can result in the compression of time suggests that top-down processes, such as explicit beliefs and expectations about causal relations, may affect the prevalence of this phenomenon. In particular, the cognitive system may take into account the causal structure of the environment, and this may moderate the extent to which causality compresses perceived time (Moore and Obhi, 2012). Support for this notion comes from studies that manipulate the salience of different types of causal mechanisms and examine the effect it has on time compression. In one study, participants first elaborated on various physical versus biological phenomena in order to prime causal forces that typically dissipate or build up over time (Faro et al., 2010, Study 2). For instance, to prime physical forces that dissipate over time, participants wrote about how a rock that is thrown into water can capsize a toy boat. To prime biological forces that build up over time, they wrote about how a person who smokes can contract a lung disease. As part of another task, participants then made elapsed time judgments for pairs of historical events. Time compression between causally related historical events was attenuated when participants had considered biological causal mechanisms before the focal task. The priming of different causal mechanisms did not have an effect for time estimates for events that were not causally related, ruling out potential anchoring on short versus long time intervals that could be evoked by the priming.

In another study, participants considered emotions (e.g., pride, anger) vs. traits (e.g., courage, arrogance) of the actors involved in the causally related historical events before making elapsed time judgments (Faro et al., 2010, Study 3). Those considering emotions—a type of causal force that is typically seen as dissipating over time—made shorter time estimates, and time compression was greater relative to the baseline condition. Ratings of whether the emotions involved in the events dissipated (vs. built up) over time were correlated with time estimates. These findings on the role of emotions in the compression effect for historical events are consistent with research showing that motivations and desires play a role in intentional binding studies that employ much shorter time intervals (Engbert and Wohlschläger, 2007).

These recent findings show that although compression of time may be driven by an automatic process, it can also be modulated by higher-level beliefs about causation. The extent to which one or the other plays a role can vary by the task. The role that different types of causal relations play in moderating the effect is also consistent with the mechanism-based view of causal reasoning (Ahn et al., 1995; Ahn and Kalish, 2000). This view proposes that when people say that A causes B, they believe that there is a process that takes place between A and B in which a force or causal power is transmitted. This approach, and the concept of force in particular, can suggest a more-specific accounting for time compression than reliance on the general relationship between time and causality (see Table 1). At least in some settings, compression may occur because people believe that many causal forces tend to dissipate, and so, for a cause to have impact, it “needs” to occur close to the effect in time (McCloskey, 1983). Thus, people might compress the time between causes and effects because they believe predominantly in dissipative causal forces, which dominate our early experiences with causality and with physical objects (Faro et al., 2010; see also White, 1998, 1999). This may be especially plausible with conceptual action-outcome sequences like historical events. It would be instructive to examine whether such variations in expectations about types of causal relations would modulate the effect in other settings.

What can we make of these findings from the different settings the effect of causality on time judgments have been observed? Our aim here is not to propose a specific process account for the phenomenon, as its various manifestations may be driven by different specific processes (see Table 1 for a summary of different explanations proposed). However, the evidence reviewed here does entail some linkages and consistent findings. The phenomenon seems to reflect a possibly automatic, unconscious response: The implicit manipulation of intentions, and the role of cognitive resources in enhancing the effect, point in this direction. That the effect persists in more-conceptual settings, with longer time intervals and with manipulations of causality that take place after the experience of the interval, suggests that the compression tendency can be abstracted and generalized to settings in which people reason more explicitly about causality. That is, people may compress time through an automatic judgment process or the brain's motor function but can draw on causal information to compress time also in more-deliberate settings. The salience of non-default, less familiar types of causal relations (e.g., with causal forces that build up over time) mutes this tendency in some settings. But the default and predominant effect of perceiving a causal relation between two events is to subjectively compress the time between them.

There is a noteworthy parallel here to the inverse and more-familiar relationship—the role that temporal contiguity plays in judgments of causality. When exposed to Michotte's phenomenological causality animations, infants as young as three months show signs of causal processing and “rely” on temporal contiguity between cause and effect. As people mature, these early, partly innate and automatic responses are generalized and play a role as cues to causality in inference (White, 1988). Cognitive development and ability make people more sensitive to temporally distant causes, and here, too, beliefs and expectations about more-complex causal mechanisms reduce the tendency to rely on temporal proximity to infer causality (Fletcher et al., 1986; Schlottmann, 1999; Hagmayer and Waldmann, 2002; Buehner and May, 2003). It is thus possible that the two responses—the effect of time on causality, and of causality on time—are rooted in a common underlying source.

## Implications

Most of the work examining the effect of intentionality or causality on time judgments has focused on its implications for the sense of agency. The adjustment of time for intentional actions and their outcomes was seen as evidence for a prereflective sense of agency, as an implicit marker of agency (see Moore and Obhi, 2012). The compression of time that resulted from the shifts in the times of occurrence of actions and outcomes was seen as an index of agency rather than as a variable of interest per se. We next discuss the implications of this work for decisions that are dependent on time judgments.

### Compression and planning of action

The subjective compression of elapsed time between actions and their effects may affect people's plans for when a given action would need to be taken to produce timely impact in the future. In a study that tests this hypothesis, participants first consumed chewing gum and then received bogus feedback that their performance on an alertness task showed improvement. Participants then learned that the chewing gum was (or was not) causally associated with improving performance on alertness tasks (Faro, 2010). As dependent measures, participants estimated how long it may have taken for the chewing gum to have an effect on their performance (if any). This was the measure by which time compression was assessed. Then they reported the latest point they would feel comfortable using the gum again before a similar task, for an assessment whether compressed estimates of elapsed time-to-onset affected future consumption plans. Then they indicated when they were ready to begin working on the task after consuming the gum the second time. First, there was a compression of time: Participants in the strong-causal-belief condition thought the product took a shorter time to have an effect on their performance. Second, they reported that they would consume the gum closer to the time of the task on subsequent consumption. Third, they waited a shorter period before starting to work on the task after they consumed the gum again. And, finally, participants' time-to-onset estimates for the initial consumption, which were compressed through the manipulation of causality, predicted future planning and consumption decisions.

Compression of time between causes and effects can therefore make people delay their future actions. People also tend to underestimate the time between causes and effects relative to the *actual* interval (Faro et al., 2005). The combination of these factors (delaying actions because of time compression and underestimation of time relative to the actual interval) implies that people may be unrealistically optimistic in initiating actions. They would end up taking previously efficacious (i.e., causal) actions too late to be effective. This pattern is similar to the “planning fallacy,” the tendency to underestimate task completion times (Buehler et al., 1994). We believe the two phenomena are related for several reasons. First, plans may be seen as a series of cause-effect scenarios (Schank and Abelson, 1977). It is thus possible that one reason people underestimate overall task completion times, committing the planning fallacy, is because they underestimate the time between cause-effect pairs making up a plan (see also Roy et al., 2005). Second, one of the main explanations for the planning fallacy involves people's taking an “internal view” of the situation. Constructing a causal scenario represents one favorable story of how the future project is likely to unfold (Kahneman and Lovallo, 1993). In a similar vein, providing a causal scenario of how one event led to another results in time compression (Faro et al., 2005). Finally, for both phenomena there appears to be a self-other difference: Underestimation of task compression time is prominent for tasks undertaken by oneself, not by others (Buehler et al., 1994). Similarly, in some studies, compression of time was found to be stronger when an action was taken by oneself rather than by another person (Desantis et al., 2011; but see Wohlschlager et al., 2003).

Hence, the perceived compression of time between causally related events may affect the timing of subsequent actions—leading to good actions being undertaken too late. This may also be related to the tendency to underestimate task completion times. As we discuss below, compression of perceived elapsed time can also affect expectations regarding the onset of the effect in the future—with people expecting it will occur earlier than they should.

### Compression, effect onset, and placebos

People sometimes report feeling the effect of product consumption (e.g., caffeine) almost instantaneously—within an unrealistically short time after consumption (e.g., Reid, 2005). Such placebo-like effects may be driven by conditioning (Stewart-Williams and Podd, 2004) or expectations of future performance (Shiv et al., 2005). The compression of time between actions and outcomes suggests a more-specific reason for expectations of unrealistically rapid consumption outcomes. The studies we reviewed showed that perceived time of voluntary actions shifts forward in time, but at the same time, their effects subjectively shift backward in time (thus, resulting in compression of time). This may be one way in which the effect of actions, including consumption of drugs or other products, may be experienced earlier in time, especially if people believe in their causal efficacy.

To our knowledge, previous work on compression of time between actions and outcomes has not shown this tendency to expect that effects of consumption will occur prematurely. Recent work did document a related consequence—how compression of time for a previous consumption episode may make people experience the effect earlier upon future consumption. In particular, the compression phenomenon implies that people may be prone to underestimate the time it took for a product to show its effect when they used it in the past if they believe in its causal efficacy. These recollections of too short a time-to-onset can alter people's subsequent consumption experiences, leading them to report prematurely rapid effects from subsequent consumption.

Participants in one study consumed chewing gum and then performed an alertness task (Faro, 2010). Those in the strong-cause condition were then led to believe that the gum was responsible for the improved performance they allegedly showed on the previous task. Those in the weak-cause condition were made aware of an additional possible influence on their performance (practice with the task). Replicating a compression effect, those in the strong-cause condition thought the product had been faster to have an effect on their previous performance. More importantly, upon second consumption and performance on a similar task, participants indicated they had experienced the effect of the product earlier. Time-to-onset for previous consumption predicted the timing of subsequent-effect onset.

These results of perceived earlier onset of the effect of an external substance link the compression phenomenon to placebo effects. The study reported above manipulated causal efficacy post experience. Work on placebos has shown that various factors can affect people's expectations of the causal efficacy of a treatment before it is administered and that this can affect the extent of the placebo effect. For instance, a given treatment is more effective when it is administered by a clinician than when it is administered by a computer (Colloca et al., 2004). Accordingly, future studies can examine whether manipulations that alter the perceived causal efficacy of external agents before the experience can affect the extent of temporal compression and result in effects that are experienced sooner in time.

### Compression and intertemporal choice

Past work has shown compression for causally related events that were experienced by participants themselves (Haggard et al., 2002; Engbert and Wohlschläger, 2007; Buehner and Humphreys, 2009; Moore et al., 2009; Ebert and Wegner, 2010) or by others in the past (Wohlschlager et al., 2003; Faro et al., 2005). Might a similar effect occur for events people anticipate in the future? Recent work suggests that time compression between causes and their outcomes can extend to anticipated events, and for events that participants expect to produce rather than actually produce (Engbert and Wohlschläger, 2007; Buehner and Humphreys, 2009; Buehner, 2012). Based on these findings, might a given time period expected to elapse between two events (e.g., “a government initiating public works” and “increased economic growth”) be viewed as shorter if a person believes the first event will cause the second? If the anticipated interval seems subjectively shorter, might people be more willing to wait if waiting entails a benefit?

One experimental paradigm through which such intertemporal preferences are examined is that of time discounting (see Frederick et al., 2009, for a review). In a typical study, a participant may be asked whether he or she would prefer to receive, say, £1000 now or £1500 one year from now. The tendency to choose the smaller-sooner amount instead of the larger-later amount is one way to assess the extent to which people discount future outcomes. Discounting studies typically employ two points in time that are void of a semantic link. If causality affects perception of anticipated time, imbuing the two points in time with meanings of cause and effect can affect discounting (see also Zauberman et al., 2009). Thus, a person might be more willing to wait one year and receive £1500 (when “increased economic growth” occurs) instead of £1000 now (when “public works begin”) if he or she believes the first event will cause the second. Similarly, and again because of compression in anticipated time, mentally simulating how the first event (“public works”) would cause the second (“increased economic growth”) may result in greater patience. Using language (e.g., causal verbs; see Talmy, 1988) that increases the perceived causal link between the events could have a similar effect on perceived time and discounting.

The notion that a causal relationship between the two future points in time can affect perceived duration and, as a result, time discounting, is linked to recent conjectures on discounting and another important semantic relationship—perceived similarity. Consider the common finding of hyperbolic discounting that for the same interval *t*, people are more patient when *t* is farther in the future than when it is near. Rubinstein (2003) suggested that this occurs because the similarity between two points in time separated by a common interval increases with the onset of the interval. Thus, 12 months is more similar to 11 months than 2 months is to 1 month. Similarity may also explain the recent findings that discount rates that are imputed when time is described using calendar dates are lower than those revealed when time is described as a delay (Read et al., 2005; see also LeBoeuf, 2006). In one study, respondents evaluated two delayed options framed either as 3 vs. 16 months or as August 29, 2003, vs. September 24, 2004. The authors suggested that 3 and 16 months are less similar to each other than the corresponding dates and thus result in greater discounting. Thus, there is evidence that similarity between two points in time might affect subjective duration judgments and time discounting. This recent evidence lends credence to the possibility that perceived causality can future duration assessments and time discounting in turn.

Recent work has also shown that an additional and potentially related variable, spatial distance, can affect the subjective judgments of duration and, in turn, time discounting. For instance, an individual in Philadelphia may perceive the same three-month duration from today to be longer when he or she is expecting to be in Los Angeles three months later than when he or she is expecting to be in New York (Kim et al., 2012). The person may thus be less patient and discount the same outcome more heavily when it is to be received in Los Angeles rather than in New York. The relationship between space and time discounting is noteworthy because something akin to causal time compression also occurs for spatial judgments: Perceived causation reduces spatial distance judgments (Buehner and Humphreys, 2010).

In summary, we conjectured that causality may affect judgment of time for action-outcome sequences that are anticipated in the future and that this may affect patience and decisions based on perceived time. We base this on recent work showing other conceptual, semantic relationships (similarity, spatial distance) affecting subjective time and patience as a result. The aforementioned variables are related. Time, similarity, and spatial distance are cues to causality (Einhorn and Hogarth, 1986; see also Trope and Liberman, 2010). Thus, various cues to causality may be influenced by causality, may affect each other, and in turn influence decisions that depend on the judged focal variable (e.g., time discounting).

### Compression, agency, and causal inference

The early findings that showed voluntary actions subjectively bind to their effects in time were interpreted as an implicit marker of agency. In particular, it was proposed that the “brain contains a specific cognitive module that binds intentional actions and their effects to construct a coherent conscious experience of agency” (Haggard et al., 2002, p. 384) and thus provides a feeling of fluent flow from actions to their effects (Haggard and Tsakiris, 2009). The effect was seen as a factor that enhances the sense of agency. But it may have implications for causal inference more generally. Marsh and Ahn (2009) noted that people sometimes may need or want to link ambiguous events as causes and effects. The authors suggested that time compression may be one mechanism that enables this to occur. Long elapsed time between cause and effect is typically a limiting factor for the emergence of causal beliefs. Binding related events in time allows people to form and hold causal beliefs that might otherwise conflict with the temporal proximity cue for causality (Einhorn and Hogarth, 1986). This may make people stick with certain courses of action and be less likely to switch to alternatives that have not “benefited” from compression (Faro, 2010). Thus, compression may reinforce the already advantageous role that temporal proximate causes enjoy in causal inference. Even when causes and effects are not very near in time, we may experience them as if they are or remember them as if they were.

## Conclusion

People subjectively compress the time that has elapsed between causes and effects. This phenomenon appears to be linked to a basic / primitive manner in which people process causality and time and to early experiences with the physical-mechanical environment. The tendency is robust and has been documented in a wide range of settings. The phenomenon has initially attracted attention as an implicit marker of agency, but it also has implications for the planning of action, intertemporal choice, and placebo effects. By shortening the perceived time elapsed between a focal action and an outcome, compression endows a focal course of action or cause with apparent advantage or privileged status. This in turn links the phenomenon back to causal inference: The effect of perceived causality on time perception may reinforce the tendency to attribute causality to temporally proximate causes.

### Conflict of interest statement

The authors declare that the research was conducted in the absence of any commercial or financial relationships that could be construed as a potential conflict of interest.

## Footnotes

^1^Some studies showing an effect of causality on time judgments have used the term “intentional binding,” referring to the shifts in the perceived times of occurrence of voluntary movements and their outcomes. Other work focused on judgments of the time interval and has used the term “compression.”
